# Advancing Ultrasound Beamforming With Deep Learning: A Comprehensive Review of Methods, Datasets, Benchmarks, and Computational Challenges

**DOI:** 10.1155/ijbi/4617573

**Published:** 2026-10-03

**Authors:** Hamza Hadri, Abderahhim Fail, Mohamed Sadik

**Affiliations:** ^1^ NEST Research Group LESE Lab ENSEM of Hassan II University of Casablanca Casablanca Morocco; ^2^ IESI Laboratory of ENSET Mohammedia Hassan II University of Casablanca Casablanca Morocco uh2c.ac.ma

**Keywords:** deep learning, modular beamforming pipeline, plane-wave imaging, ultrasound beamforming

## Abstract

Ultrasound imaging is a widely used diagnostic tool, and beamforming techniques are integral to its performance. Traditional methods such as delay‐and‐sum (DAS) and delay‐multiply‐and sum (DMAS) have limitations in terms of image quality and computational efficiency. Recently, deep learning approaches have shown significant promise in improving ultrasound beamforming, offering enhanced image resolution and quality, alongside potential for real‐time processing. This review is aimed at providing a comprehensive overview of the current state‐of‐the‐art in deep learning–based ultrasound beamforming. It focuses on analyzing various deep learning models, their applications, challenges, and the integration of hardware optimization strategies to enhance performance and efficiency. The review examines a range of deep learning models applied to ultrasound beamforming, including convolutional neural networks (CNNs), generative adversarial networks (GANs), transformer‐based models, and hybrid approaches. It also discusses the challenges associated with dataset limitations, model interpretability, and the risk of overfitting. Furthermore, the review explores hardware acceleration using field‐programmable gate arrays (FPGAs), along with cloud‐edge frameworks for real‐time inference. The findings highlight the potential of deep learning models to outperform traditional methods in terms of image quality and resolution. However, challenges such as the need for large and diverse datasets, the black box nature of deep learning models, and the risk of overfitting remain. Hardware optimization through FPGAs has proven effective in enabling real‐time processing, but the integration of edge computing with cloud‐based solutions offers promising avenues for balancing performance, latency, and energy efficiency. Deep learning–based ultrasound beamforming has great potential to advance medical imaging. Future efforts should enhance model generalizability with diverse and synthetic data, optimize hardware for real‐time use, and establish standardized validation protocols to support clinical adoption.

## 1. Introduction

Ultrasound beamforming has become a fundamental technique in medical imaging, offering several advantages over other imaging modalities such as x‐ray, MRI, and CT scans [1]. Unlike ionizing radiation‐based methods like x‐rays or CT scans, ultrasound imaging is noninvasive and safer for repeated use, making it ideal for continuous monitoring. In comparison with MRI, which can be expensive and less accessible, ultrasound devices are more affordable and portable, allowing for point‐of‐care diagnostics in a variety of clinical settings [2]. Furthermore, ultrasound imaging enables real‐time visualization, providing immediate feedback for dynamic processes such as blood flow and fetal development [3]. These benefits, combined with its wide applicability in areas like cardiology, obstetrics, and musculoskeletal assessment, underscore the pivotal role of ultrasound imaging in modern healthcare.

The beamforming process is the backbone of the ultrasound imaging system, playing a crucial role in determining image quality and resolution [4, 5]. Beamforming, particularly in the receiving part of the system, focuses the reflected ultrasound waves from various tissues to form coherent and high‐resolution images. By precisely aligning the signals received from different transducer elements, beamforming maximizes the signal‐to‐noise ratio (SNR), leading to clearer and more detailed images. One of the critical objectives of beamforming is minimizing sidelobes [6], which are unwanted off‐axis signals that can distort the image. Effective beamforming reduces these sidelobes, thereby enhancing image lateral resolution and reducing artifacts. This is especially important in clinical settings where accurate visualization of small structures and anomalies is essential. Reducing artifacts also improves diagnostic reliability, making beamforming a key component in delivering high‐resolution ultrasound imaging [7].

Over the years, various beamforming algorithms have been developed to improve the quality and efficiency of ultrasound imaging [8]. The most widely used and simplest approach is delay‐and‐sum (DAS) beamforming. In DAS, time delays are applied to the signals from different transducer elements to align the echoes, and then the delayed signals are summed to form the final image [9]. Although DAS is computationally efficient and easy to implement, it is prone to high sidelobe levels due to the fixed apodization weights applied, which lead to limited resolution.

To overcome the limitations of traditional beamforming, more advanced techniques have been introduced, such as delay‐multiply‐and‐sum (DMAS) [10] and minimum variance (MV) [11], which improve image quality by enhancing signal coherence and suppressing noise. DMAS introduces a nonlinear step by multiplying pairs of delayed signals before summing them, whereas MV dynamically adjusts apodization weights to optimize resolution. These methods offer superior performance over traditional approaches but increase computational demands [8].

Although advanced beamforming techniques like DMAS and MV enhance image resolution and contrast, they come with significant computational demands. The nonlinear processing in DMAS and the dynamic apodization adjustments in MV require extensive processing power and memory, potentially limiting the feasibility of real‐time imaging in clinical settings where immediate feedback is crucial [12]. Thus, although these methods improve image quality, their implementation may be constrained by the available hardware capabilities.

Other advanced algorithms, such as synthetic aperture beamforming [13] and coherence‐based beamforming [14], enhance image quality by utilizing additional signal information. Synthetic aperture methods improve spatial resolution by simulating a larger aperture through data collection from multiple transducer positions. In contrast, coherence‐based beamforming capitalizes on the coherence properties of received signals to enhance image quality, especially in challenging conditions.

Recent advancements in deep learning offer promising improvements in ultrasound beamforming techniques [4, 15]. This approach is especially advantageous in ultrasound B‐mode imaging, where traditional beamforming methods often find it challenging to balance image quality with computational efficiency. Deep learning facilitates the direct mapping of nonbeamformed data to high‐quality ultrasound images, allowing for advanced processing that uncovers complex relationships often missed by traditional algorithms [9–11]. It also optimizes the balance between image quality and computational efficiency, enabling real‐time processing of high‐resolution images. Integrating deep learning into ultrasound beamforming enhances diagnostic capabilities while addressing hardware constraints [16, 17]. The purpose of this review article is to provide a comprehensive overview of the current applications of deep learning in ultrasound beamforming. We are aimed at exploring the latest trends and ongoing research in this field, highlighting the methods and benchmarks used to evaluate the performance of deep learning–based beamforming techniques. Additionally, this review will discuss the challenges and limitations associated with integrating deep learning into ultrasound imaging, addressing both computational and clinical hurdles. Our goal is to offer insights into the potential of deep learning to advance ultrasound beamforming while acknowledging the obstacles that remain.

### 1.1. Review Methodology

This review was conducted as a narrative, expert‐guided synthesis rather than a formally registered systematic review; nonetheless, a structured search and selection process was followed to reduce selection bias and to make the scope of coverage transparent to the reader.

#### 1.1.1. Search Strategy and Sources

Relevant literature was identified through searches of IEEE Xplore, PubMed, Scopus, Web of Science, and Google Scholar, supplemented by manual screening of the reference lists of retrieved articles (backward snowballing) and of papers citing key benchmark works such as the Challenge on Ultrasound Beamforming With Deep Learning (CUBDL) challenge papers (forward snowballing). Search queries combined terms related to the imaging modality (“ultrasound”, “plane‐wave imaging”, “B‐mode”), the processing task (“beamforming”, “channel data reconstruction”, “image reconstruction”), and the modeling approach (“deep learning”, “convolutional neural network”, “generative adversarial network”, “transformer”, “self‐supervised”, “unsupervised”). Searches also covered adjacent topics required for a complete treatment of the field, namely simulation frameworks (Field II, k‐Wave, FEM/COMSOL), open benchmark datasets (Plane‐Wave Imaging Challenge in Medical Ultrasound [PICMUS] and CUBDL), and hardware acceleration (field‐programmable gate array [FPGA], application‐specific integrated circuit [ASIC], and edge/cloud computing).

#### 1.1.2. Date Range

The search targeted peer‐reviewed and archival work published between January 2015 and mid‐2025, capturing the period from the earliest deep‐learning‐based beamforming studies through the most recent transformer and hardware‐oriented approaches. A small number of earlier foundational references (e.g., the original DAS/DMAS formulations and the Field II simulator) were included for background context regardless of publication date.

#### 1.1.3. Inclusion and Exclusion Criteria

Studies were included if they (i) applied a machine learning or deep learning model to any stage of the ultrasound beamforming or channel‐data‐to‐image reconstruction pipeline, (ii) reported at least one quantitative image‐quality metric (e.g., contrast ratio [CR], contrast‐to‐noise ratio [CNR], generalized contrast‐to‐noise ratio [gCNR], peak signal‐to‐noise ratio [PSNR], and structural similarity index [SSIM]) or a clearly described qualitative comparison against a deterministic baseline, and (iii) were published in a peer‐reviewed journal or a recognized conference proceeding (e.g., IEEE International Ultrasonics Symposium [IUS] and MICCAI). Studies were excluded if they addressed deep learning applications elsewhere in the ultrasound pipeline unrelated to beamforming or channel‐data reconstruction (e.g., pure image classification or segmentation of already‐beamformed B‐mode images without a reconstruction component), if they were not available in English, or if they were preprints that had not undergone any peer review.

#### 1.1.4. Venue Considerations

Deep learning–based ultrasound beamforming is a field in which several of its most influential contributions, including the CUBDL challenge itself and the majority of its top‐performing submissions, were disseminated as IEEE IUS conference papers rather than journal articles, since IUS functioned as the challenge′s dedicated venue; excluding this literature would omit foundational results rather than improve rigor. Where a given contribution was later extended into a peer‐reviewed journal article (e.g., the CUBDL evaluation framework itself [18]), the journal version is cited preferentially and the original conference paper is cited only where it reports results (such as individual challenge submissions) not superseded by the journal version. Foundational architecture papers that are conventionally cited via their arXiv preprints even in the published literature (e.g., VGGNet [19] and MobileNet [20]) are retained in that form for consistency with standard citation practice in the field; the reference list is now predominantly composed of peer‐reviewed journal articles, with conference proceedings retained specifically where they are the original or only source for a result discussed in the text.

## 2. Background

### 2.1. Ultrasound Beamforming

Ultrasound beamforming is not a single operation but comprises two distinct stages, *transmit beamforming* and *receive beamforming*, which together shape both the outgoing acoustic wave and the incoming echo signal. In transmit beamforming, the system applies element‐specific time delays (and, optionally, apodization weights) to the excitation pulses sent to each transducer element so that the emitted wavefronts constructively interfere at a chosen transmit focus (in focused imaging) or form a specific wavefront shape, such as a plane or diverging wave (in unfocused, high‐frame‐rate imaging) [4, 21]. Once the transmitted wave is backscattered by tissue and captured by the array, receive beamforming applies a second, typically much finer, set of per‐channel time delays, apodization weights, and a summation (or other combination) rule to the received channel data to reconstruct a focused image line or region. Because receive delays can be continuously updated as a function of depth, receive beamforming supports dynamic focusing throughout the field of view, whereas transmit focusing is fixed for a given transmit event. Figure 1 illustrates this receive‐side chain; the algorithms discussed in the remainder of this section, DAS, DMAS, and MV, are all *receive* beamforming algorithms that operate after transmission and echo reception, and it is at this receive stage that essentially all of the deep learning methods reviewed later in this paper are applied.

**Figure 1:**
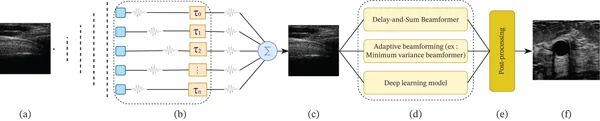
The receive‐side ultrasound beamforming chain that follows wave transmission and echo backscatter: (a) acoustic echo reception at the transducer array, (b) per‐element signal capture, (c) application of per‐channel receive (dynamic‐focusing) time delays, (d) apodized beamforming summation across channels, (e) signal postprocessing (envelope detection, log compression, scan conversion), and (f) final B‐mode image generation. Panels (b)–(e) together constitute the receive beamforming stage; the transmit beamforming stage that precedes echo reception is described separately in the text below.

A critical aspect of ultrasound receive beamforming is the challenge of balancing image quality with frame rate [21], especially in real‐time imaging applications. Beamforming algorithms differ significantly in their approach to signal processing and apodization, which refers to the weighting of signals from transducer elements to control the beam shape and reduce artifacts [22, 23].

In DAS beamforming (see Figure 2), per‐channel receive delays are continuously updated with depth to provide dynamic receive focusing, and apodization weights (e.g., a Hanning or Tukey window across the active aperture) are commonly applied and may themselves be updated dynamically (e.g., expanding‐aperture apodization) as the focal depth changes [9]. In many practical DAS implementations, however, the apodization profile is comparatively simple and depth‐invariant in shape even when the aperture size is dynamically adjusted, which limits its ability to suppress sidelobes compared with the fully data‐adaptive weighting used in MV beamforming (described below). DAS is computationally efficient and enables faster frame rates, but its reliance on a fixed, data‐independent apodization profile (rather than one derived from the statistics of the received data) leaves comparatively higher sidelobe levels, which impacts image clarity and lateral resolution [9].

(1) Ix,y=∑k=1nWk·Skt−τk.

**Figure 2:**
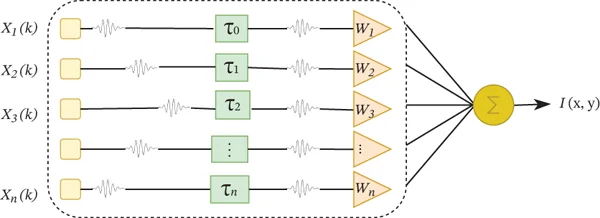
Schematic representation of the delay‐and‐sum (DAS) beamforming algorithm [9]. This diagram illustrates the core steps in the DAS beamforming process, where signals from multiple transducer elements are delayed by specific time shifts (*τ*_1_, *τ*_2_. ⋯ , *τ*_*n*_) and weighted by corresponding apodization factors (*w*_1_, *w*_2_, *w*_*n*_) before being summed together to form a coherent beam.

The equation for DAS beamforming is expressed in Equation (1), where *I* (*x*, *y*) is the intensity (or image value) at the point (*x*, *y*) in the image, *n* is the number of transducer elements, *W*_*k*_ is the apodization weight applied to the *k*‐th transducer element′s signal, *S*_*k*_(*t*) is the signal received by the *k*‐th transducer element at time *t*, and *τ*_*k*_ is the delay applied to the *k*‐th transducer element′s signal so that echoes from the focal point arrive in phase across all elements. DMAS (see Figure 3) was originally proposed for microwave breast cancer imaging and later adapted to ultrasound B‐mode imaging by Matrone et al. [24], who reformulated its processing steps specifically for ultrasound signals. Rather than summing the individually delayed channel signals directly as in DAS, DMAS first forms the pairwise products of every combination of two delayed channel signals; because this pairwise multiplication is a nonlinear (second‐order) operation, it doubles the signal bandwidth and shifts part of the resulting spectrum outside the original transducer passband. Consequently, a full DMAS implementation involves additional processing steps beyond the multiplication itself: each pairwise product is typically passed through a band‐pass filter centered on the original fundamental frequency to reject this out‐of‐band content (giving rise to the commonly used “filtered DMAS” or F‐DMAS formulation [24]), and a signed square‐root (or similar amplitude‐normalizing) operation is applied to restore the original signal units before the products are summed. It is this combined sequence, pairwise multiplication, band‐pass filtering, signed square‐rooting, and summation, that constitutes DMAS as used in practice, rather than pairwise multiplication alone. As with DAS, DMAS relies on dynamic, depth‐dependent receive delays; unlike DAS, however, its principal contrast and sidelobe‐suppression gains come from this nonlinear combination of channel pairs rather than from the receive apodization profile, which is typically comparable to that used in DAS. The pairwise combination step increases computational complexity from *O*(*n*) for DAS to *O*(*n* ^2^) for a direct implementation of DMAS, which significantly limits achievable frame rates in real‐time imaging settings.

**Figure 3:**
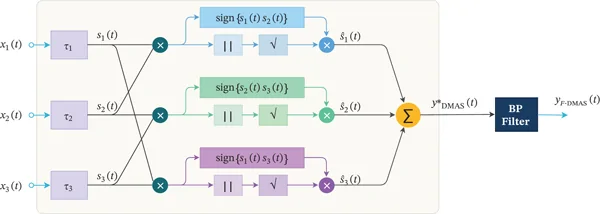
Schematic representation of the filtered delay‐multiply‐and‐sum (F‐DMAS) beamforming algorithm [24] for a 3‐channel configuration. Channel signals *x*_*i*_(*t*) are aligned via delays *τ*_*i*_, combined via signed square‐root pairwise multiplication to maintain dimensionality, summed, and passed through a band‐pass (BP) filter to produce the final signal y_F−DMAS_ (*t*).

Because of this computational cost, a substantial body of literature has focused on reformulating DMAS for reduced complexity and real‐time deployment. Balanced or reduced‐complexity variants include the double‐stage delay‐multiply‐and‐sum (DS‐DMAS) formulation of Mozaffarzadeh et al. [25], which re‐expresses part of the pairwise expansion in terms of a DAS‐like inner combination to further suppress sidelobes without a proportional increase in cost, and pixel‐based, Wiener filter–combined formulations [26] that trade a controlled amount of contrast gain for reduced processing overhead. On the implementation side, several studies have demonstrated real‐time or near‐real‐time DMAS beamforming through GPU and hybrid CPU–GPU acceleration, using vectorized reformulations of the nested pairwise summation to exploit data‐parallel hardware [27]. Collectively, this literature indicates that the raw O(*n*_2_) complexity of DMAS is not by itself prohibitive for real‐time use once these algorithmic and hardware‐level optimizations are applied, but it does mean that, absent such optimization, DMAS remains substantially more demanding than DAS, which motivates much of the interest in deep learning–based approximations of DMAS‐ and MV‐like image quality discussed later in this review (Section 4).

Ix,y=∑k=1n−1∑m=k+1nsgnskt−τk·smt−τmskt−τk·smt−τm.

The equation for DMAS beamforming (Equation (2)) combines the delayed signals from *n* transducer elements, where *S*_*k*_(*t*) and *S*_*m*_(*t*) are the signals received by the *k*‐th and *m*‐th transducer elements at time *t*, and *τ*_*k*_ and *τ*_*m*_ represent the delay times applied to these two elements. The outer sum runs over *k* = 1, …, *n* − 1 and the inner sum over *m* = *k* + 1, …, *n*, so that every distinct pair of transducer elements (*k*, *m*) with *k* < *m* is combined exactly once. The signed square‐root operation is applied to each pairwise product to restore the original signal amplitude units after multiplication, as described above; in a full F‐DMAS implementation, a band‐pass filtering step (omitted from the equation for brevity, following common practice in the literature) is applied to each pairwise product before this signed square‐root step [24].

### 2.2. Ultrasound Deep Beamforming

Deep learning, a branch of artificial intelligence, brings a new dimension to ultrasound beamforming by mapping nonbeamformed data (see Figure 4) directly to beamformed images [28]. This approach allows for sophisticated statistical processing of raw RF signals or demodulated IQ data, enabling the model to learn intricate relationships that conventional deterministic algorithms may overlook. Deep learning can dynamically optimize factors like apodization and signal coherence, enhancing both image resolution and frame rate. By addressing the trade‐off between image quality and computational efficiency, deep learning–based beamformers enable real‐time processing and rendering of high‐resolution images without overwhelming hardware constraints, significantly improving diagnostic capabilities [29].

**Figure 4:**
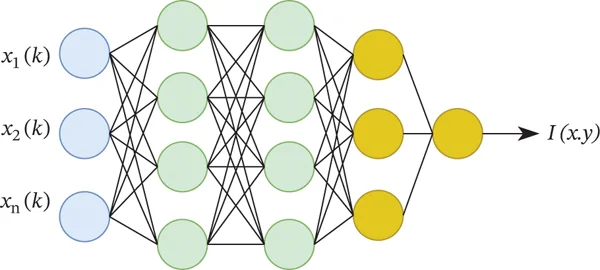
A simplified, generic neural network architecture illustrating the deep learning–based beamforming paradigm described in [28]. The input layer represents the signals from transducer elements, followed by hidden layers of neurons that learn to model complex relationships and enhance signal coherence. The output layer provides the final beamformed ultrasound data; specific architectures (CNN, GAN, transformer, etc.) elaborating on this general paradigm are presented in Section 4.

One of the earliest applications of deep learning in ultrasound beamforming replaced traditional methods with a U‐Net, processing RF channel data directly to segment features like anechoic cysts. This approach [30] improved accuracy, minimized the need for multiple insonification angles, and enhanced noise suppression, marking a key milestone in demonstrating the feasibility of deep learning for ultrasound beamforming.

In addition to beamforming, deep learning can be utilized for complete image reconstruction, mapping RF raw data directly to B‐mode images. However, this approach presents its own challenges, including the need for large datasets and effective training strategies to ensure accuracy. These topics will be discussed in further detail later in the paper.

## 3. Deep Learning–Based Ultrasound Beamforming

The primary aim of this survey is to illustrate the state of the art in applying deep learning techniques to ultrasound beamforming. This encompasses a thorough examination of recent advancements in beamforming algorithms, and the influence of various approaches on overcoming existing challenges and limitations in the field of ultrasound imaging. To make comparison across the very heterogeneous literature tractable, we organize the state‐of‐the‐art section around a taxonomy defined by the dominant architectural paradigm employed (fully connected, convolutional, generative‐adversarial, unsupervised/self‐supervised, transformer‐based, or dual/cascaded models), and, within each subsection, we explicitly compare studies in terms of (i) the stage of the pipeline being learned (channel‐data weighting, image‐to‐image refinement, or full reconstruction), (ii) the training data regime (simulated, phantom, in vivo, paired vs. unpaired), and (iii) the reported gains relative to DAS or MV baselines. A synthesized cross‐architecture comparison is provided at the end of Section 4 and in Table 1.

**Table 1:** Summary of deep learning techniques for ultrasound beamforming.

Reference	Objective	Method	Dataset	Evaluation metrics	Key findings
[31]	Suppress sidelobes, enhance contrast, and noise suppression.	DNN with feed‐forward multilayer architecture.	Simulated, phantom, and in vivo data	CR and contrast‐to‐noise ratio (CNR)	Outperformed DAS with improved contrast ratio (CR) and CNR across all datasets.
[32]	Reconstruct high‐resolution images from subsampled data.	Fully convolutional network (DeepFormer)	Subsampled raw ultrasound data	SSIM and PSNR	Comparable results with fully sampled data, offering an efficient alternative to traditional beamforming techniques.
[33]	Reconstruct high‐quality ultrasound images using fewer RF channels.	27‐layer convolutional neural network (DeepBF)	Subsampled RF data.	CNR, gCNR, PSNR, and SSIM	High‐quality images generated across various subsampling rates and detector configurations, outperforming traditional DAS beamforming.
[34]	Enhance beamforming quality by transforming DAS signals into higher quality MV outputs.	Convolutional autoencoder (CAE), fully connected network (FCN), and U‐Net.	Simulated datasets.	Signal‐to‐noise ratio (SNR).	Improved image quality by reducing noise and enhancing SNR, with faster processing than traditional MV beamforming methods.
[35]	Improve image quality of single plane wave ultrasound using deep learning–based channel compounding.	1D fully convolutional network with pixel‐wise weighting (CUBDL 1st‐place).	Simulated phantom data (CIRS Model 054GS) and PICMUS dataset.	MSE, MS‐SSIM.	Produced higher quality images than classical single‐angle beamforming, showing improved sharpness and artifact reduction.
[36]	Approximate minimum variance beamforming (MVB) to speed up image reconstruction.	Modified MobileNetV2 architecture with depth‐wise separable convolution (CUBDL 2nd‐place).	Public datasets from the UltraSound ToolBox (USTB).	Visual comparison with DAS and MVB.	Achieved image quality similar to MVB with much lower computational time, improving practicality for mobile ultrasound devices.
[37]	Enhance image quality and reduce computational complexity for ultrafast ultrasound imaging.	FCN for preliminary beamforming and CAE for image refinement.	Simulations, phantom, and in vivo data.	Contrast and resolution.	Achieved comparable image quality to MV beamforming with significantly reduced computational time, enabling real‐time frame rates (1000 FPS).
[38]	Enhance ultrasound imaging by reducing grating lobe artifacts in sparse linear arrays.	CNN adapted from U‐Net with residual connections, multiscale, and multichannel layers.	Simulated dataset with undersampled arrays and high‐quality reference images.	Mean signed logarithmic absolute error.	Improved image quality by eliminating GL artifacts while preserving critical details and speckle patterns, enabling more efficient and cost‐effective ultrasound imaging.
[39]	Improve resolution and reduce artifacts in single plane‐wave ultrasound imaging.	Modified U‐Net with EfficientNet‐B5 as the encoder and U‐Net as the decoder.	Simulated and experimental RF data.	Resolution, CNR, and noise suppression.	Achieved substantial improvements in resolution, CNR, and noise suppression over traditional DAS and some deep learning approaches.
[40]	Achieve superresolution imaging directly from Full Matrix Capture (FMC) data (NDT application).	Multiscale residual U‐Net (FMC‐Net) with encoder–decoder structure and skip connections.	Simulated and experimental FMC data.	Structural similarity and defect‐localization accuracy relative to TFM ground truth.	Demonstrated superior performance over traditional methods (TFM and wavenumber algorithm) by effectively visualizing subwavelength defects and enabling real‐time ultrasonic imaging deployment.
[41]	Reconstruct high‐quality ultrasound images from sub‐Nyquist channel data.	U‐Net–based encoder–decoder model optimized with a custom loss function (mean − squared logarithmic error + structural similarity).	Multiorgan scans with subsampled Fourier measurements.	Mean‐squared logarithmic error and structural similarity.	Achieved high‐resolution, high‐contrast images comparable with MV beamforming with significantly reduced data volume and computational cost.
[42]	Achieve high‐quality ultrasound imaging from single plane‐wave acquisitions, matching diverging wave imaging quality.	Complex Residual Attention U‐Net (C‐Res‐Att‐UNet) with complex convolutions and attention mechanisms.	Simulated, in vitro, and in vivo datasets.	Contrast, resolution, structural similarity.	Improved image quality by leveraging both phase and magnitude information, achieving high frame rates suitable for real‐time applications.
[43]	Integrate ultrasound beamforming, image denoising, and subsampling into a single multitask model.	CNN with six convolutional blocks and task‐specific weight normalization.	CUBDL challenge datasets and diverse ultrasound data.	Contrast, resolution, and noise reduction.	Outperformed traditional DAS methods in pixel‐level metrics, showing improved noise reduction and high‐quality reconstruction from single‐angle acquisitions.
[44]	Enhance image quality by approximating an ideal point spread function (PSF) in single‐plane wave imaging.	U‐Net with wavelet transforms in the encoder and decoder, step‐by‐step loss function progression (MSE to L0.2).	Simulated and in vivo RF datasets.	Axial resolution, lateral resolution, and contrast.	Achieved substantial improvements in resolution and contrast over DAS beamforming, demonstrating robustness and effectiveness for real‐time high‐quality ultrasound imaging without fine‐tuning.
[45]	Enhance resolution in single‐plane wave ultrasound imaging while preserving high frame rate.	WSRGAN with a U‐shaped generator using DWT/IDWT and an adaptive wavelet attention module (AWAM).	PICMUS dataset.	FWHM, contrast, PSNR, and SSIM.	Significantly improved imaging metrics (FWHM, contrast, PSNR, and SSIM), outperforming conventional methods like DAS and other deep learning–based superresolution techniques.
[46]	Enhance ultrasound image quality using a multiconstrained GAN‐based beamforming approach.	MC‐HGAN with RF‐based intrinsic learning, image‐based perceptual learning, and a fusion module.	Datasets with various ultrasound emission modes.	PSNR, SSIM, SNR, and CR	Outperformed conventional and deep learning–based beamformers in image clarity and computational efficiency, producing high‐resolution and high‐contrast images.
[47]	Improve ultrasound image quality through unsupervised deconvolution without requiring paired training data.	DeepDeconv using a cycle‐consistent GAN framework with U‐Net generator and LS‐GAN discriminator.	Simulated and in vivo data with subsampled RF at various downsampling rates.	PSNR, SSIM, and CR.	Achieved significant improvements in contrast, resolution, and PSNR, producing quality close to supervised models without fine‐tuning, suitable for portable ultrasound devices.
[48]	Improve ultrasound imaging quality without ground‐truth data using coherence‐based learning.	Deep coherence learning (DCL) with a U‐Net–inspired CNN and normalized coherence loss.	Simulation, phantom, and in vivo data.	Resolution and contrast.	Demonstrated superior resolution and contrast compared with DAS and DMAS, offering a robust unsupervised approach for high‐frame‐rate, high‐quality single plane‐wave imaging.
[49]	Improve image resolution and contrast with minimal computational overhead for resource‐limited devices.	Tiny‐VBF, a ViT‐based encoder–decoder model with transformer blocks and multihead attention layers.	In vitro and in silico datasets.	Resolution and contrast.	Demonstrated significant improvements over DAS and Tiny‐CNN methods. Deployed on FPGA using a hybrid quantization scheme, reducing resource consumption by over 50% while maintaining image quality.
[50]	Enhance single‐plane wave ultrasound imaging with high frame rate and limited data.	Progressively dual reconstruction network (PDRN) with progressive and dual networks for beamforming.	Simulated, in vivo, and CUBDL datasets.	CNR and PSNR.	Demonstrated improved image quality metrics (CNR and PSNR) even with limited training data, enabling adaptation to unpaired data and addressing rapid tissue motion.
[51]	Reconstruct high‐quality ultrasound images from single plane‐wave RF data while reducing dependency on transmission angles.	Cascaded deep neural network with PixelNet for pixel weighting and cGAN for image refinement.	PICMUS and CPWC datasets (training), CUBDL dataset (testing).	Resolution and noise suppression.	Demonstrated strong generalization and robustness on unseen data, enabling faster frame rates compared with multiangle coherent compounding methods.

### 3.1. Early Attempts at Deep Learning–Based Beamforming

Early explorations into the use of deep neural networks (DNNs) for ultrasound beamforming were focused on improving image quality by suppressing off‐axis scattering. One study [31] investigated a DNN‐based approach that operated in the frequency domain using the short‐time Fourier transform. By training different networks for various frequencies, the method is aimed at enhancing ultrasound channel data through suppression of sidelobes and improvement of contrast and noise ratios, which was demonstrated across simulated, phantom, and in vivo data. The DNN approach consistently outperformed traditional DAS beamforming, with notable improvements in CR and CNR observed in both simulations and real‐world scenarios.

The networks employed in this approach used real and imaginary components of the signals as inputs, with a feed‐forward multilayer architecture. Notably, dropout techniques and optimized activation functions like ReLU were crucial for preventing overfitting and enhancing training performance. The success of these early DNN implementations laid the groundwork for the expanded use of deep learning methods in ultrasound beamforming, demonstrating their potential to revolutionize the field by offering enhanced image resolution and adaptability.

The emergence of convolutional neural networks (CNNs) [19, 20, 52], in image segmentation tasks has revolutionized various fields, including medical imaging. Their ability to learn complex patterns and features from data enabled precise localization and classification of structures within images.

This success has paved the way for the application of these networks in ultrasound beamforming. One notable work [54] explored using a deep CNN (see Figure 5), based on U‐Net [55], and VGG‐13 encoder [19] to extract essential image details directly from raw radiofrequency (RF) channel data, rather than relying on traditional beamforming techniques. The study utilized Field II simulations [56] to train the network on single plane wave (PW) insonification data of cysts in tissue, producing segmentation masks that accurately located targets of interest.

**Figure 5:**
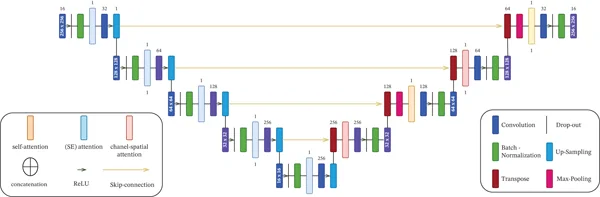
Block diagram of the U‐Net convolutional neural network proposed by [53], featuring squeeze‐and‐excitation (SE), self‐attention, and channel‐spatial attention modules at multiple scales.

In [32], the authors introduced DeepFormer, a deep learning–based architecture that reconstructs high‐resolution ultrasound images from subsampled raw data. By leveraging a fully convolutional network, DeepFormer captures correlations between neighboring scan lines, enabling the generation of high‐quality images with reduced computational effort. The method showed comparable results to fully sampled data, offering an efficient alternative to traditional beamforming techniques.

In [33], the authors also utilize subsampled RF data to address the limitations of traditional DAS beamforming in ultrasound imaging. They propose a deep learning–based beamformer, DeepBF, which employs a 27‐layer CNN architecture to generate high‐quality images across various subsampling rates and detector configurations. Similar to DeepFormer [32], the method is aimed at reconstructing high‐quality ultrasound images using fewer RF channels and employing a deep convolutional network to handle sparse data. Relative to DeepFormer, DeepBF targets a wider range of subsampling and detector configurations and reports gains in gCNR in addition to CNR and PSNR, making the two studies complementary rather than directly comparable on identical benchmarks.

The study in [34] investigates three deep learning models: a convolutional autoencoder (CAE), a fully connected network (FCN), and a U‐Net to enhance ultrasound beamforming. The models were trained on simulated datasets to transform noisy DAS signals into higher quality MV outputs. The networks improved image quality by reducing noise and enhancing SNR while also being faster than traditional MV beamforming methods; among the three, the U‐Net variant was reported to give the best trade‐off between accuracy and inference speed, illustrating an early instance of the accuracy/latency trade‐off that recurs throughout this review.

The success of CNN architectures, particularly U‐Net, in accurately segmenting ultrasound images marked a shift towards deep learning as a promising approach in ultrasound beamforming.

The use of another deep learning modality, self‐supervised learning, has been explored in various domains, including ultrasound beamforming. In the work by Zhang et al. [57], the authors model the inverse problem [58] by mapping initial low‐quality images to high‐quality outputs through a DNN, using contrastive loss and regularizations tailored for ultrasound data. This approach improves image quality, robustness, and computational efficiency in beamforming and, unlike the fully supervised CNN approaches discussed above, does not require paired high‐quality reference images during training.

### 3.2. CUBDL Challenge‐Winning Architectures

One of the significant milestones in advancing deep learning techniques for ultrasound beamforming is the CUBDL [18, 59], a public benchmark competition described fully in Section 5.4, together with its dataset composition and evaluation infrastructure. The challenge attracted widespread participation [35, 36, 60, 61], and the discussion below focuses specifically on the architectures of the two winning submissions, which outperformed traditional DAS beamforming in key performance areas such as image resolution and contrast.

The winners of the CUBDL challenge [35, 36] introduced deep learning techniques that successfully generated high‐quality ultrasound images from a single PW, comparable to those created using multiple PWs. The first‐place entry [35] utilized a 1D CNN architecture to generate pixel‐wise weights for the beamforming process. Additionally, the authors found that simpler 1D neural network architectures achieved results comparable to more complex 2D and 3D models tested in their ablation experiments, despite lower computational demands.

The second‐place entry [36] proposed a deep learning method for ultrasound image reconstruction from prebeamformed channel data, employing a modified MobileNetV2 architecture to approximate minimum variance beamforming (MVB). Their approach includes preprocessing steps such as IQ demodulation and time delay compensation, followed by training on a diverse dataset from phased and linear array probes. MobileNetV2′s efficient depth‐wise separable convolutions enable rapid generation of beamformed data components, which are refined into B‐mode images through envelope detection. As reflected in Figure 6 and Table 1, both winning entries relied on relatively lightweight, single‐plane‐wave‐input architectures rather than deep 3D CNNs, which is consistent with the challenge organizers′ finding that architectural complexity beyond a certain point yielded diminishing returns on the CUBDL benchmark [18].

**Figure 6:**
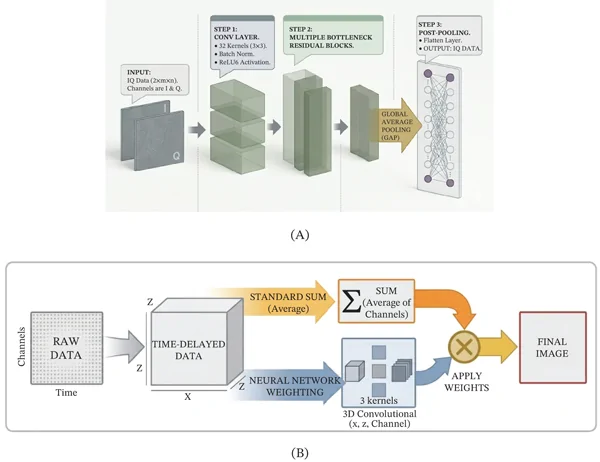
Architectures of the top two CUBDL challenge submissions: (A) the first‐place entry by Rothlübbers et al. [35], a 1D convolutional neural network that generates pixel‐wise beamforming weights from channel data; and (B) the second‐place entry by Goudarzi et al. [36], a modified MobileNetV2 architecture that approximates minimum variance beamforming.

## 4. State of the Art in Ultrasound Deep Learning Based Beamforming

### 4.1. Fully Connected Network Approach

Wang et al. [37] propose the ABF‐MV network, which combines adaptive beamforming with deep learning to enhance image quality in ultrafast ultrasound imaging. The model is aimed at replicating MVB performance but with reduced computational complexity, thus improving the frame rate and suitability for real‐time applications.

The ABF‐MV network consists of two components: a FCN for preliminary beamforming and a CAE for final image refinement. The FCN performs a nonlinear summation of channel RF data, generating preliminary scanline data. The CAE then refines this data by encoding it into a sparse representation and decoding it into high‐quality RF scanline data. Unlike MV beamforming, which calculates complex apodization weights with covariance matrix inversions, ABF‐MV simplifies this by learning data‐driven transformations that approximate MV results.

The authors validate ABF‐MV through simulations, phantom, and in‐vivo data, demonstrating that it achieves comparable contrast and resolution to MV beamforming but with a significantly lower computational burden. Testing also shows that ABF‐MV can deliver real‐time frame rates at around 1000 FPS, making it a promising option for ultrafast ultrasound imaging. Because the fully connected stage still requires one output neuron per scanline sample, this approach scales less favorably to large aperture or 3D imaging than the convolutional approaches discussed next, which share weights spatially.

### 4.2. CNN Approach

CNNs have become a prominent approach in ultrasound beamforming due to their ability to efficiently capture spatial patterns and complex relationships in imaging data. CNNs address the challenges of ultrasound beamforming by learning optimal filters directly from data, enabling the model to extract features that enhance resolution, contrast, and overall image quality. Through stacked convolutional layers, CNNs can map the raw ultrasound signals to high‐quality, beamformed images. These networks can capture both local and global contextual information, allowing for adaptive beamforming that responds dynamically to varying imaging conditions. In recent studies, CNN‐based approaches have outperformed traditional methods in producing sharper, clearer images, particularly when handling noise and artifacts typical of medical ultrasound. Within this broad family, the studies below can be further grouped by the specific bottleneck they target: sparse/undersampled array data ([38]), single‐plane‐wave resolution recovery ([39, 44]), nonmedical/FMC imaging ([40]), sub‐Nyquist data volumes ([41]), complex IQ‐domain processing ([42]), and multitask learning across several of these objectives simultaneously ([43]).

The paper in [38] presents a CNN‐based approach to enhance ultrasound imaging with sparse linear arrays, addressing the challenge of grating lobe (GL) artifacts that typically arise from undersampling. The proposed method involves two primary steps: First, a low‐quality initial estimate of the ultrasound image is generated using a DAS algorithm on undersampled data from single PW insonifications; second, a CNN specifically trained to reduce diffraction artifacts is applied to this DAS output to reconstruct a higher quality image. The CNN architecture, adapted from U‐Net with residual connections, employs multiscale and multichannel convolutional layers to effectively eliminate GL artifacts while preserving critical image details and speckle patterns. Training was conducted on a simulated dataset using low‐quality inputs from undersampled arrays and corresponding high‐quality reference images, with a mean signed logarithmic absolute error loss to ensure consistency with the dynamic range and ultrasound image properties. This approach demonstrated strong potential to improve image quality for ultrasound systems using sparse arrays, potentially enabling more efficient and cost‐effective ultrasound imaging.

The CNN‐based beamformer used in [39] presents a modified U‐Net architecture for ultrasound image reconstruction, specifically targeting single plane‐wave data. To overcome the resolution and artifact challenges of single‐wave imaging, the model uses EfficientNet‐B5 as the encoder and U‐Net as the decoder, creating a robust deep learning–based beamformer that processes pre‐beamformed RF data. Training data consist of simulated RF data from scatterers with varying intensities, augmented with random and exponential gain adjustments to mimic tissue attenuation. The model was evaluated using both simulated and experimental data, achieving substantial improvements in resolution, CNR, and noise suppression over traditional DAS and even some other deep learning approaches.

In [40] the authors proposed FMC‐Net, a multiscale residual U‐Net for superresolution ultrasonic phased array imaging directly from Full Matrix Capture (FMC) data. FMC‐Net leverages an encoder–decoder structure with multiscale residual modules and skip connections to model complex wave interactions, enabling high‐resolution image reconstruction. Trained on simulated and experimental data, FMC‐Net demonstrates superior performance over traditional methods like the total focusing method (TFM) and the wavenumber algorithm, effectively visualizing subwavelength defects and achieving superresolution imaging. Notably, this study originates from the nondestructive testing (NDT) literature rather than medical imaging (see Section 6.3), illustrating the cross‐pollination between the two domains. This approach shifts computational load to the training phase, allowing efficient, real‐time ultrasonic imaging deployment.

In ultrasound beamforming, sub‐Nyquist sampling reduces data and computational requirements by acquiring fewer samples than the Nyquist rate while retaining key imaging details. The paper in [41] introduces a deep learning–based method for reconstructing high‐quality ultrasound images from sub‐Nyquist channel data. By using subsampled Fourier measurements from a sparse subset of channels, the proposed approach reduces data volume and computational cost while maintaining image quality. The authors employ a U‐Net–based encoder–decoder model trained on multiorgan scans to reconstruct B‐mode images directly from partial data. The model is optimized using a custom loss function combining mean‐squared logarithmic error and structural similarity, yielding high‐resolution, high‐contrast images comparable to MVB but at a fraction of the data requirements. This method shows promise for low‐cost, efficient ultrasound imaging that is both hardware‐ and data‐efficient.

The Complex Residual Attention U‐Net (C‐Res‐Att‐UNet) proposed in [42] targets fast ultrasound imaging from single plane‐wave acquisitions, aimed at matching the quality of diverging wave imaging. By incorporating complex convolutions and attention mechanisms in the complex domain, C‐Res‐Att‐UNet leverages both phase and magnitude information of complex in‐phase/quadrature (IQ) data, improving image quality by focusing attention on key image regions. The network comprises custom complex layers, including complex concatenation, max pooling, and upsampling, to preserve complex data structure. Evaluated on simulated, in vitro, and in vivo datasets, this approach achieves high contrast, resolution, and structural similarity, approaching the quality of coherent compounding while maintaining high frame rates suitable for real‐time applications.

In [43], the paper presents a multitask approach for ultrasound beamforming, employing a CNN to simultaneously address multiple ultrasound imaging tasks. Their method integrates ultrasound beamforming with image denoising and subsampling within a single model, optimizing both for high frame rate and image quality.

The proposed architecture consists of six convolutional blocks, each containing two convolutional layers activated by GELU. This network is capable of handling various tasks by applying a unique weight normalization scheme, where scale and bias parameters are adjusted for each task (beamforming, speckle noise reduction, and channel data subsampling). The approach allows for flexibility, as the CNN can adapt its weights for different task requirements without the need for multiple separate models.

The model is trained and evaluated on a diverse set of ultrasound data, showing that it outperforms traditional DAS methods in pixel‐level metrics, particularly for noise reduction and high‐quality reconstruction from single‐angle acquisitions. The authors validate their approach using CUBDL challenge datasets, demonstrating improvements in contrast and resolution.

Deep reconstruction to beamforming in single PW imaging is presented in [44], which enhances image quality by approximating an ideal point spread function (PSF). This method reconstructs a high‐quality tissue reflectivity function from RF data.

The authors′ model adopts a U‐Net structure with wavelet transforms in the encoder and decoder to maintain high‐frequency details. This architecture processes RF channel data to approximate a high‐quality PSF modeled as a 2D Gaussian kernel. To enhance training, a step‐by‐step loss function approach is used, progressing from coarse to fine levels by adjusting the loss function from mean squared error (MSE) to finer L0.2 constraints. This approach effectively resolves limitations in typical PWI, which often suffers from poor resolution and contrast due to the wide main‐lobe and sidelobes of traditional beamforming methods.

Testing on simulated and in vivo datasets showed substantial improvements in axial and lateral resolution and contrast when compared with DAS beamforming. The approach, validated on datasets without fine‐tuning, highlights its robustness and effectiveness for real‐time, high‐quality ultrasound imaging.

Taken together, the CNN‐based studies above show a consistent pattern: gains in contrast and resolution over DAS are achieved fairly reliably across architectures, but the amount of independent, out‐of‐distribution (OOD) validation varies widely, from single‐dataset evaluations (e.g., [38]) to broader validation on CUBDL and multiorgan data (e.g., [41, 43]), which should be taken into account when comparing the reported numbers across studies.

### 4.3. Generative Adversarial Network (GAN) Approach

The authors of [45] propose WSRGAN, a GAN‐based method for enhancing ultrasound imaging resolution, specifically for single PW images. Traditional plane‐wave ultrasound imaging methods like CPWC improve image quality by synthesizing multiple beams, but they compromise the high frame rate of single‐beam imaging. WSRGAN is aimed at overcoming this by directly superresolving low‐resolution single‐beam images, preserving frame rate while enhancing image quality (see Figure 7).

**Figure 7:**
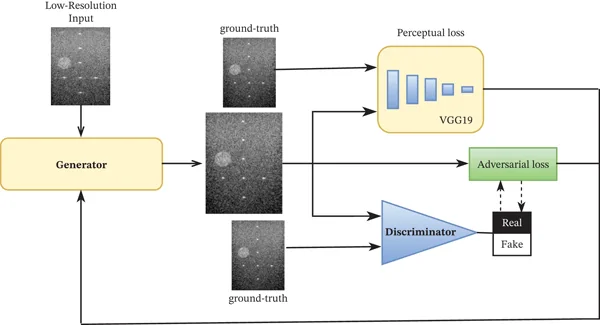
Schematic of a generic superresolution GAN (SRGAN‐style) architecture, illustrating the generator and discriminator networks and the composite loss function combining adversarial loss and a VGG19‐based perceptual loss. The WSRGAN [45] and MC‐HGAN [46] models discussed in this subsection follow this same generator‐discriminator‐perceptual‐loss paradigm, with WSRGAN replacing the generator′s pooling/upsampling layers with wavelet‐domain operations and MC‐HGAN introducing an additional RF‐domain branch and fusion module.

The core of WSRGAN′s architecture includes a U‐shaped generator network that employs the discrete wavelet transform (DWT) and inverse discrete wavelet transform (IDWT) instead of conventional pooling and sampling layers, effectively preventing information loss in the wavelet decomposition process. An adaptive wavelet attention module (AWAM) further enables the model to focus on important high‐ and low‐frequency image details selectively, enhancing specific features in the reconstruction process. Additionally, a novel wavelet‐based GAN loss, combined with SSIM and perceptual loss, guides the adversarial training, allowing WSRGAN to achieve high‐quality image reconstruction with minimal artifacts.

Experiments on the PICMUS dataset demonstrate that WSRGAN significantly improves imaging metrics such as full width at half maximum (FWHM), contrast, PSNR, and SSIM, outperforming conventional methods like DAS and other deep learning–based superresolution techniques. The MC‐HGAN model proposed in [46] is a beamforming approach that combines GANs with multiple constraints to enhance ultrasound image quality. The MC‐HGAN framework comprises three main modules: an RF‐based intrinsic learning module, an image‐based perceptual learning module, and a fusion module that combines the outputs of the first two. The RF‐based module learns from RF signal data to capture essential beamforming features, whereas the image‐based module learns from coarse ultrasound images to improve perceptual quality. A multiconstrained training strategy, incorporating RF constraints, image constraints, and a GAN‐based adversarial loss, guides the model to produce high‐resolution and high‐contrast images.

The authors validate MC‐HGAN on datasets with different ultrasound emission modes, demonstrating that it outperforms conventional and deep learning–based beamformers in terms of both image clarity and computational efficiency. Compared with WSRGAN, MC‐HGAN′s dual RF/image branches make it better suited to settings where raw channel data is available at training time, whereas WSRGAN operates purely as a post‐beamforming image‐domain superresolution step and is therefore more portable across acquisition hardware.

### 4.4. Unsupervised and Self‐Supervised Learning Approaches

Combining a GAN with an unsupervised framework was implemented in [47]. This paper introduces DeepDeconv, an unsupervised deep learning approach designed to improve ultrasound imaging quality by deconvolution without requiring paired training data. Traditional deconvolution techniques in ultrasound imaging face challenges due to high computational costs and the need for an accurate PSF. DeepDeconv addresses these issues by learning a mapping between low‐ and high‐quality image domains using only unpaired data, making it suitable for portable and low‐power ultrasound devices.

The network architecture employs a cycle‐consistent GAN framework, where a generator network (U‐Net) reconstructs high‐resolution images, and a discriminator network validates image quality. For unsupervised learning, a cycle‐consistency loss enforces accurate reconstruction across both domains, and LS‐GAN is used to refine realism in the outputs. The DeepDeconv model is trained on simulated and in vivo data, achieving substantial improvements in contrast and resolution for subsampled RF data at various downsampling rates. Quantitative evaluations show that DeepDeconv significantly enhances PSNR, SSIM, and CR, achieving quality close to supervised models without fine‐tuning.

In [48], Cho et al. propose deep coherence learning (DCL) to improve ultrasound imaging quality without ground‐truth data. DCL uses a normalized coherence loss to measure and optimize coherence between signals within a set of PW data, which enables the model to retain coherent signals (like main lobes) and suppress artifacts (e.g., sidelobes and GLs). This unsupervised training approach avoids the limitations of supervised methods that depend on high‐quality reference images, making DCL versatile in clinical imaging contexts.

DCL is implemented with a U‐Net–inspired CNN architecture that processes 2D complex baseband I/Q data. The model′s encoder–decoder structure, with skip connections, learns features from each PW in the set to generalize beamforming for various probe configurations. During training, coherence loss is calculated between the model′s prediction and the target frames within the PW set, enforcing coherence adaptively. When tested on simulation, phantom, and in vivo data, DCL demonstrated superior resolution and contrast compared with traditional methods like DAS and DMAS, marking it as a robust approach for high‐frame‐rate, high‐quality single‐PW imaging. Compared with DeepDeconv, DCL avoids the domain‐mismatch risk inherent to unpaired cycle‐consistent training, since its coherence objective is derived directly from the physics of the acquired PW set rather than from an auxiliary unpaired high‐quality image domain; this makes DCL′s supervisory signal more directly traceable to the input data, at the cost of being specific to multi‐PW acquisition protocols.

### 4.5. Recurrent and Sequence‐Based Approaches

Compared with CNN‐ and GAN‐based methods, recurrent neural networks (RNNs) and other sequence models have seen more limited direct application to the core beamforming task, but they are relevant wherever ultrasound data is treated as a temporal or sequential signal rather than a static 2D image. Because channel data across transducer elements, or successive frames in ultrafast plane‐wave sequences, can be viewed as ordered sequences, RNN‐family architectures (including LSTM and GRU variants) and sequence‐to‐sequence formulations have been explored as an alternative to convolutional processing for capturing temporal dependencies between transducer elements or between successive compounded frames [5]. In practice, most RNN‐based work in this space treats beamforming as a sequence‐to‐sequence mapping from per‐element or per‐angle channel samples to an output scanline, which can capture element‐to‐element dependencies that a purely convolutional receptive field may only approximate. However, the sequential (nonparallelizable) nature of standard recurrent inference makes RNNs less attractive than CNNs or transformers for real‐time deployment, which likely explains their comparatively sparse adoption relative to convolutional and transformer‐based architectures in the recent literature; this remains an open area with room for further investigation, for instance via lightweight or parallelizable recurrent units.

### 4.6. Transformer Approach

In [49], Rahoof et al. propose Tiny‐VBF, a vision transformer (ViT)–based model. Tiny‐VBF is aimed at improving image resolution and contrast with minimal computational overhead, making it suitable for deployment on resource‐limited devices. The Tiny‐VBF model adopts an encoder–decoder architecture with transformer blocks that incorporate multihead attention layers. Initially, time‐of‐flight‐corrected RF data are fed into the encoder, which extracts key features from the ultrasound data. These features are then reconstructed in the decoder to produce a beamformed IQ‐demodulated image. The model uses a MSE loss function to guide training, with MVDR‐beamformed IQ data as the ground truth. The authors validate Tiny‐VBF on in vitro and in silico datasets, where it demonstrates substantial improvements in contrast and resolution compared with both DAS and Tiny‐CNN methods.

Tiny‐VBF is deployed on an FPGA using a hybrid quantization scheme that reduces resource consumption by over 50% while maintaining image quality. The FPGA architecture includes processing elements that handle efficient computations with on‐chip BRAM for optimized data handling. Testing across bit‐widths confirmed that hybrid quantization preserves resolution and contrast, enabling Tiny‐VBF to achieve high‐quality beamforming on resource‐constrained hardware. Unlike the CNN‐ and RNN‐based approaches above, the self‐attention mechanism in Tiny‐VBF gives it a global receptive field from the first layer, which is reported to help resolve the wide main‐lobe/sidelobe trade‐off discussed in Section 2, but at a nominally higher computational cost that the authors mitigate specifically through quantization and FPGA deployment.

### 4.7. Dual/Cascaded Model Approach

Using two models in sequence for the beamforming process is another approach adopted by some works, such as [50]. Gao et al. introduce the progressively dual reconstruction network (PDRN), a deep learning model designed to enhance single PW ultrasound imaging through a combination of progressive and dual networks. This model addresses the challenge of high‐frame‐rate imaging with limited image quality by reconstructing high‐quality images from minimal PW data.

The PDRN consists of two main components: a progressive network that incrementally learns beamforming data for increasingly compounded PWs and a dual network that reconstructs beamformed data with reduced wave counts, adding constraints that reduce the learning space and improve model accuracy. This dual network also enables the model to adapt to unpaired data, facilitating transfer learning to address cases where ground‐truth data is insufficient, especially in situations with rapid tissue motion. Experiments using simulated, in vivo, and CUBDL datasets validate PDRN′s effectiveness, showing that it improves image quality metrics like CNR and PSNR, even when trained with limited data.

The authors of [51] propose a robust cascaded DNN for ultrasound image reconstruction from single PW RF data, using two specialized networks: PixelNet and a conditional generative adversarial network (cGAN). The approach begins by transforming RF data at different angles into a common 0° data representation, reducing the network′s dependence on PW transmission angles. PixelNet, a fully convolutional network, then calculates optimal pixel weights for the DAS‐beamformed image, enhancing quality by suppressing artifacts. The DAS image is further refined through the cGAN, which improves resolution and reduces noise. This two‐step model allows high‐quality image generation from single‐angle acquisitions, enabling faster frame rates compared with multiangle coherent compounding methods. The model’s performance was evaluated using PICMUS and CPWC datasets for training, with the CUBDL dataset reserved for testing, showing strong generalization and robustness on unseen data. Both dual/cascaded approaches share the design principle of separating a coarse, fast reconstruction stage from a learned refinement stage, but PDRN cascades two learned networks end‐to‐end for a single acquisition protocol, whereas [51] cascades a deterministic‐style pixel‐weighting network with a generative refinement network and explicitly cross‐validates on an independent benchmark (CUBDL) not seen during training, which represents a comparatively stronger test of generalization among the studies reviewed in this section.

### 4.8. Cross‐Architecture Comparison

Across the six architectural families reviewed above, a few trends stand out. First, nearly all approaches report improvements over DAS in at least one contrast‐ or resolution‐related metric, but comparability across studies is limited by the use of different datasets, region of interest (ROI) definitions, and metric subsets (Table 1); this is one of the reasons a standardized benchmark such as CUBDL (Section 3.2) is valuable. Second, there is a broad trade‐off between model expressiveness and deployability: fully connected and lightweight CNN models (e.g., MobileNetV2‐based approaches and Tiny‐VBF) tend to prioritize real‐time and edge feasibility, whereas GAN‐based and dual/cascaded models tend to prioritize maximum image quality at higher computational cost. Third, the training‐data regime varies considerably, from fully supervised approaches that require paired DAS/MV or high‐quality reference images, to self‐supervised and unsupervised approaches (Section 4.4) that relax this requirement at some cost in training stability or model specificity. These trends motivate the dataset and hardware‐focused discussions in Sections 5 and 6.

## 5. Dataset

In deep learning–based ultrasound beamforming, data quality and diversity are foundational to model effectiveness, as deep learning is inherently a statistical approach that relies on data distributions to learn meaningful patterns. Unlike traditional beamforming methods, which primarily leverage deterministic signal processing algorithms, deep learning models require extensive datasets to capture the underlying variability within ultrasound signals. High‐quality data across diverse conditions such as different tissue types, patient demographics, imaging angles, and transducer settings enables these models to generalize well and produce accurate beamformed images across varying clinical contexts. Without sufficiently diverse datasets (simulation, experimental, in vivo), models may overfit to particular conditions or fail to account for the full spectrum of variability present in real‐world ultrasound imaging. Therefore, assembling datasets from varied sources is crucial to achieving robust and generalizable deep learning beamformers that meet clinical demands for accuracy and adaptability.

### 5.1. Field II and UltraSound Toolbox (USTB)

The Field II ultrasound simulation program [56, 62] is a powerful tool widely used in ultrasound research for generating realistic datasets, essential for training and validating deep learning–based beamforming models. Developed to simulate both single and multielement ultrasound transducers, Field II can handle complex scenarios, such as synthetic aperture imaging, by calculating spatial impulse responses for various probe configurations and apodization schemes [63]. This allows researchers to generate highly detailed synthetic ultrasound datasets, modeling conditions that span a wide range of clinical settings. The toolbox supports linear wave propagation within homogeneous media, enabling controlled, reproducible experiments that help ensure deep learning models are exposed to a diverse array of acoustic scenarios. These datasets play a crucial role in the statistical training of deep learning algorithms, supporting their ability to generalize effectively [64, 65]. With Field II, it is possible to simulate both the emitted and received ultrasound fields, creating data that closely mimics real‐world conditions, from tissue scattering to advanced beamforming techniques.

The USTB [66] is a MATLAB‐based framework designed for processing and comparing ultrasound imaging techniques, including various beamforming algorithms, across diverse datasets. In the context of deep learning, USTB′s primary utility is in beamforming raw RF data using advanced algorithms to produce high‐resolution images. These high‐quality images serve as ground truth for training and validating deep learning–based beamformers.

### 5.2. Computational Simulation Frameworks for Ultrasound Beamforming

Computational modeling plays a fundamental role in the development and validation of ultrasound beamforming techniques [67]. In addition to experimental acquisitions, numerical simulation tools enable controlled investigations of acoustic wave propagation, transducer characteristics, and tissue interactions. These tools are particularly important in the context of deep learning–based beamforming, where large and diverse datasets are required for model training and validation. Beyond spatial impulse response simulators such as Field II, several advanced numerical frameworks based on finite‐difference time‐domain (FDTD) and finite element method (FEM) formulations are widely used in ultrasound research [68, 69].

#### 5.2.1. FDTD‐Based Simulation Tools

FDTD [68] methods solve the full acoustic wave equation directly in the time domain. Unlike spatial impulse response approaches that assume linear propagation in homogeneous media, FDTD‐based solvers can model heterogeneous, nonlinear, and frequency‐dependent acoustic phenomena.

One of the most widely adopted open‐source FDTD toolboxes in ultrasound research is *k-Wave* [70]. This MATLAB‐based framework enables the simulation of time‐domain acoustic wave fields under realistic conditions, including nonlinear propagation, power‐law absorption, and complex boundary conditions. The flexibility of FDTD formulations allows accurate modeling of wave interactions in heterogeneous tissues, making them suitable for high‐intensity focused ultrasound (HIFU) [71], harmonic imaging, and advanced beamforming validation. FDTD approaches provide higher physical fidelity compared with simplified linear models. However, this accuracy comes at the cost of increased computational complexity, requiring substantial memory and processing power, especially for three‐dimensional simulations.

#### 5.2.2. FEM‐Based Simulation Tools

The FEM [69] offers another powerful framework for ultrasound modeling, particularly when dealing with complex geometries and multiphysics interactions. FEM discretizes the computational domain into finite elements and solves the governing equations using variational formulations. Commercial multiphysics platforms such as *COMSOL Multiphysics* [72] enable coupled simulations involving acoustic propagation, structural mechanics, and piezoelectric effects. These capabilities are especially valuable for transducer design optimization, acoustic lens modeling, and fluid‐structure interaction analysis. FEM‐based approaches can also handle anisotropic and layered media more naturally than some FDTD implementations.

Despite their flexibility and precision, FEM simulations are computationally intensive and are generally more suitable for system design and physical modeling rather than large‐scale dataset generation for deep learning applications.

#### 5.2.3. Comparison of Simulation Frameworks

Different simulation tools serve complementary roles in ultrasound beamforming research. Spatial impulse response simulators such as Field II provide efficient modeling of linear wave propagation and are widely used for algorithm validation. In contrast, FDTD and FEM frameworks offer full‐wave modeling capabilities at the expense of computational cost, as summarized in Table 2.

**Table 2:** Comparison of computational simulation frameworks for ultrasound beamforming.

Method	Example tool	Physics modeled	Typical runtime ^∗^	Main strength	Limitation
Spatial impulse response	Field II	Linear propagation, homogeneous media	Seconds to minutes per frame	Fast, efficient beamforming validation; standard in the DL beamforming literature	Assumes linear, homogeneous media; no nonlinear or multiple‐scattering effects
FDTD	k‐Wave	Nonlinear propagation, power‐law absorption, heterogeneous media	Minutes to hours per 2D frame; hours to days in 3D	Full‐wave and nonlinear modeling; suitable for HIFU and harmonic imaging	High computational and memory cost, especially in 3D
FEM	COMSOL	Coupled acoustic–structural–piezoelectric physics	Minutes to hours, depending on mesh size	Multiphysics and transducer/lens design modeling; handles anisotropic media	Computationally intensive; less suited to large‐scale dataset generation
Processing Framework	USTB	Not a wave propagation simulator; postprocesses channel data	Seconds per frame	Standardized beamforming benchmarking and ground‐truth generation	Requires externally acquired or simulated channel data as input

^∗^Approximate, hardware‐ and configuration‐dependent figures reported across the cited literature; intended to illustrate relative, not absolute, computational cost.

#### 5.2.4. Relevance to Deep Learning–Based Beamforming

In deep learning–based ultrasound beamforming, simulation frameworks fulfill two essential roles: (1) generation of synthetic training datasets and (2) controlled benchmarking of reconstruction algorithms. High‐fidelity FDTD and FEM models enable the creation of physically realistic datasets that capture heterogeneous tissue properties and nonlinear acoustic effects, potentially improving model generalization.

However, due to their computational demands, large‐scale dataset generation using full‐wave solvers remains challenging. Hybrid strategies may therefore be adopted, where limited high‐accuracy simulations are combined with data augmentation or generative modeling techniques to expand dataset diversity while maintaining physical consistency.

Overall, the integration of advanced computational simulation frameworks significantly strengthens the methodological foundation of deep learning–based ultrasound beamforming research and supports the development of robust and clinically transferable models.

### 5.3. PICMUS

The PICMUS dataset [73] was established to standardize and evaluate ultrasound beamforming techniques, particularly for plane‐wave imaging applications. The dataset consists of four distinct subsets: two simulated and two experimental. The simulated datasets, generated with Field II, include isolated scatterers and anechoic cysts over a speckled background, each with 75 plane‐wave transmissions covering angles from −16° to 16°. The experimental datasets, acquired using a Verasonics Vantage 256 system on a phantom, provide similar setups with scatterers and cysts for direct comparisons. Each dataset supports both RF (raw modulated) and IQ (demodulated) formats, enabling comprehensive testing of beamforming algorithms across various data processing conditions.

In deep learning–based beamforming, the PICMUS dataset is invaluable for training and validation, providing a consistent ground truth [74, 75]. The high‐quality, high‐resolution images obtained from these datasets allow for objective comparison of beamforming techniques on metrics like contrast, resolution, and speckle appearance, making PICMUS a cornerstone in advancing ultrasound imaging methods through deep learning.

Despite its widespread adoption, PICMUS has notable limitations that should be considered when interpreting results obtained on it. The dataset is restricted to a small number of phantom geometries (point targets and anechoic cysts) and a single acquisition system, so models validated exclusively on PICMUS may not generalize to the anatomical complexity, tissue heterogeneity, and probe diversity encountered in vivo, a form of domain shift that is rarely quantified in the papers reviewed here. Furthermore, because PICMUS images are typically compared against coherently compounded (multiangle) DAS as a reference, this reference is itself an imperfect, DAS‐derived pseudo‐ground‐truth rather than an independent measure of true tissue reflectivity; models trained or evaluated against it may therefore be implicitly rewarded for reproducing DAS‐like artifacts or biases rather than for improving true diagnostic accuracy.

### 5.4. CUBDL

The CUBDL [59] represents a pivotal initiative aimed at redefining ultrasound beamforming through advanced deep learning approaches. CUBDL focused on leveraging neural networks to directly process raw RF data and reconstruct high‐quality ultrasound images. By providing standardized datasets, including both simulated phantoms and in vivo acquisitions, the challenge facilitated consistent evaluation of novel methodologies [18]. Its emphasis on performance metrics such as resolution, contrast, and computational efficiency established CUBDL as a benchmark platform for advancing beamforming research.

CUBDL provided an extensive and diverse set of datasets [76, 77] to facilitate the benchmarking and evaluation of deep learning–based beamforming methods. The main dataset for Task 1 consisted of 21 image acquisition sequences from six institutions worldwide. These included a mix of simulated, phantom, and in vivo data, acquired using multiple ultrasound systems and transducers with varying frequencies (3.1–8 MHz) and sampling rates (6.25–78.125 MHz). Phantom data featured targets like wires and cysts, some with ex vivo tissue layers for added acoustic clutter, whereas in vivo data captured the brachioradialis muscle. Postchallenge contributions enriched the dataset with additional in vivo breast ultrasound data from six patients and phantom data from multiple models, expanding the total database to 576 sequences. These datasets incorporated diverse acquisition settings, ensuring variability and robustness for testing. CUBDL assembled the largest open‐access international ultrasound channel database, enabling fair evaluations and supporting the development of generalized deep learning models for ultrasound beamforming.

The CUBDL challenge produced open‐source evaluation code implemented in Python using PyTorch. This code benchmarks deep learning–based beamforming methods, offering tools for image quality evaluation (e.g., contrast, resolution, and SNR) and sound speed correction via speckle brightness maximization. It includes pre‐trained network weights from top submissions, enabling reproducibility and fair comparisons. The framework supports benchmarking against ground truth images, promoting standardization and transparency in ultrasound beamforming research.

Despite these strengths, CUBDL also has limitations that temper its use as a universal benchmark. The in vivo component remains anatomically narrow, centered mainly on the brachioradialis muscle and, in later extensions, the breast, so anatomical diversity (e.g., cardiac, fetal, abdominal, or vascular imaging) is not well represented. As with PICMUS, the reference images used for quantitative comparison are themselves produced by conventional (typically multiangle coherent compounding or MV) beamformers, so CUBDL scores primarily measure similarity to another beamformer′s output rather than to an independent measure of tissue truth; this pseudo‐ground‐truth dependence means a model could in principle achieve a high CUBDL score by closely mimicking DAS/MV‐specific artifacts. Finally, CUBDL, like PICMUS, does not define standardized clinical endpoints (e.g., lesion detectability by a radiologist, diagnostic agreement, or downstream task performance), so improvements in CR, CNR, gCNR, or PSNR on these benchmarks do not by themselves establish clinical benefit, and, as discussed further in Section 6, a model can improve image‐similarity metrics while altering diagnostically relevant speckle statistics or subtle lesion boundary appearance in ways these metrics do not capture.

### 5.5. Performance Metrics

The evaluation of B‐mode images produced by deep learning–based ultrasound beamformers is a critical aspect of their development and validation [78]. Accurate assessment ensures that these models achieve high‐quality imaging, a prerequisite for reliable clinical diagnosis and treatment planning. Metrics used to evaluate beamformed images serve as objective measures to quantify various aspects of image quality, including resolution, contrast, and noise suppression [79, 80]. In addition, these metrics facilitate a direct comparison between traditional and advanced beamforming techniques, highlighting the improvements introduced by deep learning models.

#### 5.5.1. CR (*C* *R*_*d* *B*_)

The CR quantifies the difference in brightness between two distinct ROIs in the image, typically a hypoechoic or anechoic target region and a surrounding background region. It is calculated as the logarithmic ratio of the mean intensities (*μ*_ROI1_ and *μ*_ROI2_) of the two regions. This metric is a localized measure, focusing on the intensity contrast between specific regions, making it particularly useful for evaluating tissue boundary visibility and differentiation:

CRdB=20·log10μROI1μROI2.

#### 5.5.2. CNR

The CNR extends contrast evaluation by incorporating noise levels, offering a normalized measure of contrast relative to the underlying image variability. It uses the mean intensities and standard deviations (*σ*_ROI1_ and *σ*_ROI2_) of the two regions, focusing on localized differences:

CNR=μROI1−μROI2σROI12+σROI22.

#### 5.5.3. PSNR (*P SNR*_dB_)

The PSNR is a global metric that evaluates overall image quality by comparing the reconstructed image (*I*_*R*_) to a reference image (*I*). It is inversely related to the MSE between the two images, making it a sensitive measure of global image fidelity:

PSNRdB=10·log10MAXI2MSEI,IR,

where MAX_I_ denotes the maximum possible pixel intensity value of the image (e.g., 255 for an 8‐bit image or 1 for a normalized image), and MSE (*I*, *I*_*R*_) is computed over an image of size *M* × *N*.

MSEI,IR=1MN∑i=1M∑j=1NIi,j−IRi,j2

#### 5.5.4. gCNR

The gCNR [81, 82] measures the separability between the intensity distributions of two ROI, and was proposed specifically to be robust to nonlinear postprocessing (e.g., dynamic range compression) that can distort conventional CNR and CR values. Denoting by *f*_1_(*x*) and *f*_2_(*x*) the empirical probability density functions of pixel intensities in ROI 1 and ROI 2, respectively, gCNR is defined as one minus the overlap (the total variation distance) between these two distributions:

gCNR=1−∫−∞∞minf1x,f2x dx.

In practice, *f*_1_ and *f*_2_ are estimated nonparametrically from the pixel intensity histograms of each ROI, and the integral is approximated by a discrete sum over histogram bins of common width *Δ* *x*:

gCNR≈1−∑xminfˆ1x,fˆ2xΔx,

where *f*_1_(*x*) and *f*_2_(*x*) are the normalized histogram counts for a common set of bins spanning the dynamic range of both ROIs. A gCNR value of 1 indicates perfectly separable (nonoverlapping) distributions, whereas a value of 0 indicates identical distributions; unlike CR and CNR, gCNR is bounded in [0, 1] and is invariant to monotonic nonlinear compression of the displayed image [81].

#### 5.5.5. SNR

The SNR quantifies the clarity of the signal in a specific region relative to noise, based on the ratio of the mean intensity (*μ*_ROI1_) to the standard deviation (*σ*_ROI1_) within that same region. This is another localized metric that is often used to evaluate the uniformity of imaging within a region:

SNR=μROI1σROI1.

## 6. Discussion

The adoption of deep learning–based statistical approaches for ultrasound beamforming has introduced significant advances in B‐mode image quality and computational efficiency. However, these methods come with inherent limitations compared with deterministic beamforming techniques such as DAS and DMAS.

### 6.1. Challenges and Limitations

Deep learning models require extensive and high‐quality datasets for training, encompassing a wide range of clinical and simulated scenarios [18, 73]. Unlike deterministic methods, which operate based on predefined mathematical rules, deep learning models are data‐driven and highly sensitive to the quality and diversity of training data. Insufficient data or datasets that fail to capture edge cases can lead to suboptimal performance and lack of generalizability in clinical applications [83].

Although deterministic methods like DAS and DMAS provide explicit and interpretable beamforming mechanisms, deep learning models function as black boxes, making it challenging to understand the rationale behind their outputs [84]. This lack of interpretability raises concerns in clinical settings where transparency and reliability are paramount for diagnostic decision‐making [85].

The statistical nature of deep learning models increases the risk of overfitting to specific datasets, especially in the absence of robust regularization techniques or sufficient cross‐validation [86, 87]. Deterministic methods, on the other hand, are inherently generalizable as they rely on established physical principles rather than data patterns.

Deep learning–based approaches can be sensitive to input noise or variations in acquisition conditions, potentially leading to degraded image quality. Traditional beamformers like DAS and DMAS, although simpler, often demonstrate greater resilience in handling noise and artifacts due to their reliance on deterministic principles. Unlike deterministic methods that remain consistent over time, deep learning models require frequent updates and retraining to incorporate new data or adapt to different imaging conditions. This dependency increases maintenance overhead and can slow the adoption of such models in routine clinical workflows.

### 6.2. Implementation and Inference Acceleration

Deploying deep learning models often demands significant computational resources, including high‐performance GPUs or specialized accelerators like FPGAs. In contrast, deterministic methods such as DAS and DMAS are computationally lightweight and can be implemented efficiently on standard hardware. The high resource requirements of deep learning can pose challenges for real‐time applications and the integration of such models into portable ultrasound devices.

FPGAs [88] have emerged as a powerful solution for accelerating deep learning models in resource‐constrained applications like ultrasound beamforming. Their reconfigurability and parallel processing capabilities allow them to efficiently handle the computational demands of neural networks while maintaining a balance between flexibility and energy efficiency. Techniques such as pruning, quantization, and tensor decomposition are particularly beneficial for reducing memory usage and computational complexity, enabling the deployment of complex models like CNNs and LSTMs in real‐time scenarios. However, challenges remain, including the limited on‐chip memory, which often necessitates external data storage and transfer, potentially impacting performance. Optimizations like batching, parallelization, and systolic array architectures are critical to overcoming these bottlenecks, ensuring low latency and high throughput. These features make FPGAs well‐suited for implementing deep learning models in ultrasound beamforming, where real‐time processing and high accuracy are paramount.

Efficient acceleration of CNNs on FPGAs is critical for real‐time ultrasound beamforming applications. Techniques like input/output channel parallelism and deep pipelining enhance computational efficiency by leveraging the inherent parallelism in CNN operations [89]. Optimizations such as loop unrolling, pipelining, and tiling reduce memory bottlenecks and improve resource utilization, addressing the challenges posed by the limited on‐chip memory of FPGAs. Simplified CNN models, such as pruned or quantized networks, further mitigate computational demands, making FPGA deployments energy‐efficient while maintaining accuracy [90]. However, resource constraints still require balancing trade‐offs in performance, energy, and memory bandwidth, particularly for complex beamforming tasks that involve high data throughput and low latency requirements. These FPGA‐focused methods enable scalable and efficient implementations of CNNs, supporting the dynamic and resource‐intensive demands of ultrasound imaging systems.

### 6.3. Ultrasound Beamforming in NDT Applications

Although ultrasound beamforming is widely recognized for its central role in medical imaging, it also plays a critical role in NDT [91] and industrial inspection. NDT applications use ultrasonic waves to detect internal defects, cracks, inclusions, and structural inhomogeneities in materials such as metals, composites, and welded structures without causing damage to the inspected object.

In industrial phased‐array ultrasonic testing (PAUT) [92], beamforming techniques similar to DAS, DMAS, and MV are employed to steer and focus acoustic beams electronically. By controlling time delays across transducer elements, it is possible to generate sectorial scans, linear scans, or focused beams for high‐resolution defect localization. The TFM [93], based on FMC data [94], is particularly prominent in NDT due to its ability to achieve high spatial resolution in defect characterization. Compared with medical ultrasound, NDT systems typically operate at different frequency ranges and must account for highly heterogeneous and anisotropic materials, such as carbon‐fiber‐reinforced polymers or multilayered welded joints. These conditions introduce strong scattering, mode conversion, and attenuation effects, which complicate beamforming and image reconstruction.

Recent advances in deep learning–based beamforming have also begun to impact NDT applications, as illustrated by FMC‐Net [40] discussed in Section 4.2. CNNs, generative models, and hybrid physics‐informed networks have been explored for defect detection, artifact suppression, and superresolution imaging in industrial ultrasound. Similar to medical imaging, deep learning models in NDT benefit from simulated datasets generated via full‐wave solvers (FDTD or FEM), particularly when experimental data are limited or costly to acquire. Despite differences in imaging targets and regulatory environments, the underlying beamforming principles remain fundamentally similar across medical and industrial domains. Both fields face challenges related to resolution enhancement, sidelobe suppression, computational efficiency, and real‐time deployment. Consequently, methodological advances in one domain often transfer effectively to the other.

The growing convergence between medical ultrasound imaging and NDT beamforming highlights the broader impact of deep learning–based reconstruction methods. Cross‐disciplinary collaboration may further accelerate innovation, particularly in areas such as high‐resolution imaging, uncertainty quantification, and hardware‐accelerated inference.

### 6.4. Future Directions

Deep learning has made significant advancements in ultrasound beamforming, improving image quality, resolution, and computational efficiency. Despite these successes, there are several promising directions for future research that could further advance the field.

#### 6.4.1. Algorithmic Innovations

Although deep learning models have outperformed traditional beamforming techniques in many scenarios [18, 33, 34, 54], combining classical signal processing methods with deep learning could lead to more robust and adaptive solutions [50, 51]. Hybrid approaches can leverage the interpretability of classical models and the feature extraction power of neural networks.

One of the main challenges in developing deep learning models for ultrasound beamforming is the reliance on large labeled datasets. Future research can focus on self‐supervised and unsupervised learning methods to reduce the dependency on labeled data [47, 48]. These methods can use intrinsic properties of the ultrasound signals to generate training labels automatically.

Integrating ultrasound with other imaging modalities such as MRI or CT in a multimodal learning framework can enhance the diagnostic capabilities of ultrasound systems. By learning from multiple data sources, models can potentially improve image reconstruction, reduce artifacts, and enhance contrast.

#### 6.4.2. Hardware Optimization

Real‐time processing remains a critical requirement in ultrasound imaging, as noted earlier. Future efforts should focus on hardware‐specific optimizations, particularly using FPGAs and ASICs to accelerate deep learning inference for beamforming. Custom hardware implementations can significantly reduce latency and power consumption, making it feasible to deploy advanced models in portable ultrasound devices [88–90].

Edge computing can play a vital role in enabling real‐time beamforming in mobile and portable ultrasound devices. By deploying lightweight deep learning models on edge devices, clinicians can access high‐quality images without relying on cloud infrastructure, improving responsiveness and reliability in point‐of‐care settings [30]. Another promising direction is developing cloud‐edge collaborative frameworks [95], where computationally intensive tasks are offloaded to the cloud, whereas edge devices handle real‐time inference. This approach ensures a balance between performance, latency, and energy efficiency.

#### 6.4.3. Dataset and Clinical Integration

The current models are often trained on limited datasets, which can hinder generalization to diverse clinical scenarios. Future work should focus on curating large‐scale datasets that include diverse patient populations, different ultrasound machine settings, and various anatomical regions, similar to CUBDL [59].

Given the difficulty of acquiring large amounts of labeled ultrasound data, synthetic data generation using simulation tools like Field II [62] can provide a valuable supplement. Advances in generative models, such as GANs, can also be used to create realistic synthetic datasets that improve model robustness. For deep learning–based beamforming solutions to gain widespread clinical adoption, they must meet stringent regulatory requirements and be evaluated with a level of rigor comparable to that expected of other AI‐based medical devices [85].

Regulatory frameworks such as the US FDA′s guidance on AI/ML–based software as a medical device and the EU Medical Device Regulation increasingly expect evidence beyond retrospective image‐quality metrics, including documented training/validation data provenance, performance across relevant subpopulations, and a defined change‐control process for models that continue to learn after deployment. Beyond dataset curation, clinical translation of deep learning beamforming will likely require: (i) systematic failure‐mode analysis, characterizing the conditions (e.g., unusual anatomy, motion artifacts, or acoustic shadowing) under which a model′s output degrades or becomes unreliable; (ii) OOD detection mechanisms that can flag inputs dissimilar to the training distribution rather than silently producing a plausible‐looking but incorrect image; (iii) uncertainty quantification methods (e.g., ensembling, Bayesian approximations, or conformal prediction) that provide the clinician with a confidence signal alongside the reconstructed image; and (iv) prospective, reader‐based studies in which radiologists or sonographers evaluate diagnostic performance on deep learning–beamformed images under blinded conditions, rather than relying solely on retrospective, reference‐image‐similarity metrics such as CR, CNR, gCNR, or PSNR.

A further consideration, largely absent from the studies reviewed here, is the preservation of diagnostically relevant speckle statistics. Because many deep learning beamformers are optimized against image‐similarity losses (e.g., MSE, SSIM, or adversarial losses) computed relative to a DAS‐ or MV‐derived reference, there is a risk that a model achieves high scores on these metrics while altering the fine‐grained speckle texture or subtly smoothing lesion margins in ways that do not affect bulk similarity metrics but could affect a clinician’s perception of tissue heterogeneity or lesion malignancy. A related and more acute concern is the possibility of hallucinated structures: because deep generative and reconstruction models can produce visually plausible output even for inputs unlike their training data, an insufficiently validated model could, in principle, synthesize an artifact that resembles an anatomical structure not actually present in the tissue, or conversely suppress a genuine but atypical finding. Establishing standardized validation protocols that explicitly test for these failure modes, alongside the reader studies and OOD detection mechanisms noted above, will be an important prerequisite for the safe clinical adoption of deep learning–based beamforming.

## 7. Conclusion

Deep learning has demonstrated significant potential in revolutionizing ultrasound beamforming, offering improvements in image quality, resolution, and computational efficiency. By leveraging advanced neural networks, these methods can provide adaptive and data‐driven solutions that surpass traditional deterministic techniques in various scenarios. However, challenges remain, including the need for large, diverse datasets, interpretability of model outputs, and robustness in clinical settings. Hardware acceleration, particularly through the use of FPGAs and ASICs, offers a promising path to overcoming the computational demands of deep learning models, enabling real‐time processing and facilitating the integration of advanced models into portable ultrasound devices. Edge computing and cloud‐edge frameworks hold the potential to balance the trade‐offs between performance, latency, and energy efficiency, thereby improving accessibility and reliability in point‐of‐care settings. Future research should focus on addressing the limitations of current models, such as improving data diversity and developing synthetic datasets, while also ensuring that deep learning–based ultrasound beamforming meets regulatory requirements for clinical adoption. By combining the strengths of deep learning with traditional signal processing methods and optimizing hardware for real‐time performance, the field of ultrasound beamforming can continue to evolve, providing clinicians with powerful, reliable, and efficient tools for medical diagnostics.

## Funding

This study was supported by the Centre National pour la Recherche Scientifique et Technique (10.13039/501100006319; UH192022).

## Disclosure

The funder had no role in study design, data collection and analysis, decision to publish, or preparation of the manuscript.

## Conflicts of Interest

The authors declare no conflicts of interest.

## Data Availability

The data that support the findings of this study are available from the corresponding author upon reasonable request.
